# A genome-wide genetic signature of Jewish ancestry perfectly separates individuals with and without full Jewish ancestry in a large random sample of European Americans

**DOI:** 10.1186/gb-2009-10-1-r7

**Published:** 2009-01-22

**Authors:** Anna C Need, Dalia Kasperavičiūtė, Elizabeth T Cirulli, David B Goldstein

**Affiliations:** 1 Center for Human Genome Variation, Institute for Genome Sciences and Policy, Research Drive, Duke University, Durham, NC 27708, USA

## Abstract

**Background:**

It was recently shown that the genetic distinction between self-identified Ashkenazi Jewish and non-Jewish individuals is a prominent component of genome-wide patterns of genetic variation in European Americans. No study however has yet assessed how accurately self-identified (Ashkenazi) Jewish ancestry can be inferred from genomic information, nor whether the degree of Jewish ancestry can be inferred among individuals with fewer than four Jewish grandparents.

**Results:**

Using a principal components analysis, we found that the individuals with full Jewish ancestry formed a clearly distinct cluster from those individuals with no Jewish ancestry. Using the position on the first principal component axis, every single individual with self-reported full Jewish ancestry had a higher score than any individual with no Jewish ancestry.

**Conclusions:**

Here we show that within Americans of European ancestry there is a perfect genetic corollary of Jewish ancestry which, in principle, would permit near perfect genetic inference of Ashkenazi Jewish ancestry. In fact, even subjects with a single Jewish grandparent can be statistically distinguished from those without Jewish ancestry. We also found that subjects with Jewish ancestry were slightly more heterozygous than the subjects with no Jewish ancestry, suggesting that the genetic distinction between Jews and non-Jews may be more attributable to a Near-Eastern origin for Jewish populations than to population bottlenecks.

## Abstract 2

A principal components analysis of genomic information showed that individuals with full Jewish ancestry formed a clearly distinct cluster from those individuals with no Jewish ancestry.

## Background

Many genetic and non-genetic lines of evidence make clear that there are differences amongst the Jewish and non-Jewish peoples of Europe. There are both specific genetic diseases (for example, Tay-Sachs) and particular mutations (for example, the breast cancer *BRCA1 *185delAG mutation) that have considerably higher incidences in Jewish populations, and both Y chromosome and mitochondrial DNA lineages show associations with Jewish heritage [1-5]. No study, however, has directly addressed the question of whether Jewish individuals form a consistently identifiable group on the basis of genetic data alone, as has been documented for other racial/ethnic groups [6]. Recently, Price *et al*. [7] showed that self-described Jewish ancestry was a major determinant of population genetic structure in European populations, but they did not address the question of whether genetic data might be able to accurately identify which individuals do and do not have Jewish ancestry. Here we investigate whether it is possible to accurately infer the degree of Jewish ancestry using only an individual's genomic information.

To address this, we considered a random sample of 611 unrelated self-described Caucasian subjects mostly residing in America who specifically reported whether they had Jewish ancestry, and if so, how many grandparents were 'Jewish'. All individuals were genotyped for approximately 550,000 polymorphic markers and we applied a principal-component-based method to describe the population genetic structure [8] of the sample. Out of the 611 subjects, 507 reported no Jewish ancestry, 55 reported 4 Jewish grandparents, 4 reported 3 Jewish grandparents, 37 reported 2 Jewish grandparents and 8 reported 1 Jewish grandparent. Of these, 23 reported that they were Ashkenazim, one reported four Sephardic grandparents, two reported three Ashkenazi and one Sephardic grandparent, and two reported two Sephardic grandparents. A further 62 provided European or Russian country-of-origin information for at least one grandparent and 14 were able to give no more information than 'European-American'.

## Results

**Figure 1:**
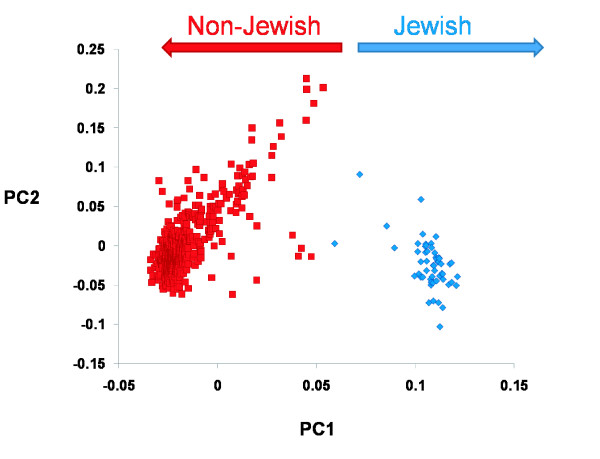
PC1 scores for Jewish and non-Jewish subjects. The score on PC1 plotted against the score on PC2 for Jewish (blue) and non-Jewish (red) subjects.

Our first test was to assess how accurately individuals with full Jewish ancestry (all four grandparents) could be distinguished from those with no Jewish ancestry using the score on the first principal component axis (PC1). We found that the individuals with full Jewish ancestry formed a clearly distinct cluster from those individuals with no Jewish ancestry (Figure 1). Strikingly, if we look only at the position on the first principal component, in this dataset, every single individual with self-reported full Jewish ancestry has a higher score than any individual with no Jewish ancestry. Interestingly, for the two subjects that appear intermediate between the clear 'Jewish' and 'Non-Jewish' clusters, one of them reports two Jewish grandparents of Sephardic origin, and one declares full Jewish ancestry, but without country of origin for their grandparents. These analyses imply the possibility of perfect or near perfect resolution of full Jewish ancestry using only genetic data. We should note, however, that if one were to attempt inference about Jewish ancestry it would be necessary to have a 'training set' such as that described here to determine the appropriate divisions between individuals with and without Jewish ancestry since the 'clusters' fall next to each other. This implies that, in practice, resolution of full Jewish ancestry would likely be less than perfect, but that the fact that we observed non-overlapping distributions on the first principle component implies that both specificity and sensitivity would be high.

**Figure 2:**
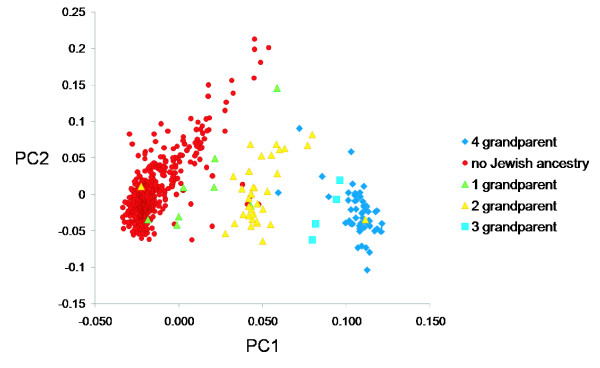
PC1 versus PC2 for people with or without Jewish ancestry. The score on PC1 plotted against the score on PC2 for people with four, three, two, one and no Jewish grandparents.

We went on to assess whether participants with one, two or three Jewish grandparents could be statistically distinguished from one another and from individuals with either full or no Jewish ancestry. As expected, most of these subjects were positioned in between the non-Jewish and the full-Jewish subjects on PC1 (Figure 2).

All but two (36/37) of the subjects with two Jewish parents scored between 0.03 and 0.08 on PC1, all four subjects with three Jewish grandparents scored between 0.08 and 0.1 on PC1, and 496/507 subjects declaring no Jewish ancestry scored below 0.3. The subjects with only one Jewish grandparent were not distinguishable based on PC1 position. The subjects that did not score within the expected range for their self-declared ancestry are shown in Table 1, along with their ancestral information where known. The majority of informative subjects with no Jewish ancestry that scored most highly on PC1 were either of Italian or Eastern Mediterranean descent. This indicates that in a mixed American context, these populations may not be easily distinguishable from subjects with a single Jewish parent.

**Table 1:** Subjects that did not score within the PC1 range expected for their self-declared Jewish ancestry group

Jewish grandparents	Expected score on PC1	Actual score on PC1	Maternal GM	Maternal GF	Paternal GM	Paternal GF
4	0.09-0.12	0.07	UK*	Unknown*	USA*	USA*
		0.06	E. Europe	E. Europe	Unknown	Unknown
2	0.03-0.08	0.11	USA	USA	USA	USA
		-0.02	UK^†^	UK^†^	Russia^†^	Russia^†^
0	<0.03	0.054	Italy	Italy	USA	USA
		0.049	E. Med.	E. Med.	USA	E. Med.
		0.048	USA	USA	Unknown	Unknown
		0.045	E. Med.^‡^	E. Med.^‡^	E. Med.^‡^	E. Med.^‡^
		0.045	USA	USA	Italy	Italy
		0.049	E. Med.	USA	USA	USA
		0.043	W. Europe^§^	W. Europe^§^	W. Europe^§^	W. Europe^§^
		0.041	Unknown	Scandinavia	W. Europe	W. Europe
		0.038	USA	USA	USA	USA
		0.032	USA	USA	USA	USA
		0.032	Italy	Italy	Italy	Italy

*Two of the grandparents are Sephardic Jews rather than Ashkenazi.^†^Country of origin is USA; UK/Russia indicates ancestry. ^‡^Specific countries not listed to prevent identification of subject, here Eastern Mediterranean indicates either Cyprus, Albania, Lebanon or Turkey; ^§^No further details known. E., east; Med. Mediterranean; W., west.

Finally, we used one-way ANOVA to determine which groups were significantly different by PC1 score from non-Jewish subjects. We found that all four groups with Jewish ancestry were significantly different, on average, from those with no Jewish ancestry: 4 versus 0 grandparents, *p *= 8.5 × 10^-256^; 3 versus 0, *p *= 4.77 × 10^-41^; 2 versus 0, *p *= 6.8 × 10^-96^; 1 versus 0, *p *= 7.8 × 10^-10^. This shows that even with only a single Jewish grandparent there remains a statistically definable signature of Jewish ancestry amongst Americans of European ancestry, although the perfect genetic discrimination of Jewish versus non-Jewish ancestry present in comparing full Jewish to no Jewish ancestry is lost at an individual level.

**Figure 3:**
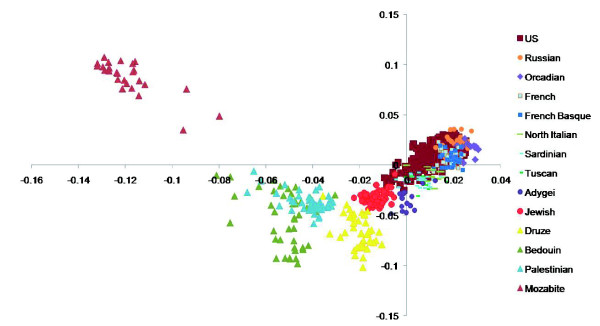
PC1 versus PC2 of Eigenstrat analysis including European and Middle Eastern subjects from the CEPH Diversity Panel. Subjects with one, two or three Jewish grandparents were excluded. Four subjects with outlying scores were excluded for better visualization of the remaining data points: a Bedouin at -0.2776,0.2012; and three Mozabites at -0.2608,0.1952; -0.2265,0.1621; -0.1611,0.1314.

To address the question of whether this axis may not be predicting Jewish, but rather (contemporary) Middle Eastern ancestry, we used the genome-wide single nucleotide polymorphism (SNP) data from the CEPH Human Genome Diversity Panel [9]. We added to our European-Americans nine other populations reflecting North Europe (Orcadian, n = 15; Central Europe (French, n = 28; French-Basque, n = 24; Northern Italian, n = 12); Southern Europe (Tuscan, n = 8; Sardinian, n = 28); and Eastern Europe (Russian, n = 25; Adygei, n = 17, an ethnic group of the Russian Caucasus). We also included Palestinian (n = 46), Druze (n = 42) and Bedouin (n = 45) samples as groups that might be similar to ancestral Jewish 'source' populations [10]. We found that the Middle Eastern populations clustered separately from the European and European-American populations, as expected, and the subjects with four Jewish grandparents clustered close to (but separate from) the Adygei and lay between the Middle Eastern and the European and European-American populations (Figure 3). This is an important finding for a number of reasons. Firstly, the Jewish subjects remain in a separate cluster when mixed with both European and Middle Eastern populations, suggesting that the original principal component axis seen in the European-Americans is indeed a Jewish-specific axis, at least in the context of the populations considered here. Secondly, the Jewish cluster lies approximately midway between the European and the Middle Eastern clusters, implying that the Ashkenazi Jews may contain mixed ancestry from these two regions. This is consistent with the Y chromosome and mitochondrial DNA genetic evidence that has been interpreted by some to suggest a stronger paternal genetic heritage of Jewish populations from the Middle East and stronger maternal genetic heritage from the host populations of the Diaspora [10]. Finally, the proximity of the Jewish cluster to the Adygei is of interest, but the small sample size of the Adygei and sparse availability of Central Asian populations makes interpretation of this proximity difficult.

## Discussion

These analyses make clear that individuals with full Jewish ancestry are a genetically distinct group from those having no (self-reported) Jewish ancestry. Of the subjects that self-identified as Jewish and knew their type, almost all were Ashkenazim. Of the Jewish subjects that did not know their type but could provide information on grandparent country of origin, the vast majority had Eastern or Central European ancestry, and none had Mediterranean or Middle Eastern ancestry. Finally, one of the two subjects reporting partial Sephardic ancestry did not cluster clearly with the other Jewish subjects. Considering also that 90% of American Jews are Ashkenazim [3], we conclude that this axis is specific to Ashkenazi Jews, and that we cannot make any conclusions about other types of American Jews (for example, Sephardic, Mizrahi) from these data.

This leaves open the question, however, of why Americans of Jewish ancestry are a distinct group. There are two extreme possibilities: either the Jewish group reflects ancestry from source populations other than those of non-Jewish Americans; or Jewish populations have undergone bottlenecks that change their genetic makeup.

These possibilities can be distinguished to a degree by comparing the position of the full Ashkenazi Jewish cluster to a series of geographically distributed populations represented by the human genome diversity panel [9]. We found that the full Jewish cluster fell between that of Middle Eastern and European populations. We also compared the average heterozygosity across the set of linkage disequilibrium-pruned polymorphisms in those with full Jewish ancestry to those without, and found that the subjects with four Jewish grandparents were, on average, slightly more heterozygous than the subjects with no Jewish ancestry. These data therefore suggest that the Jewish group is distinguished from non-Jewish Europeans more because of their genetic heritage in the Near East than due to population bottlenecks perturbing the genetic composition of Jewish groups.

## Conclusion

We show that, at least in the context of the studied sample, it is possible to predict full Ashkenazi Jewish ancestry with 100% sensitivity and 100% specificity, although it should be noted that the exact dividing line between a Jewish and non-Jewish cluster will vary across sample sets, which, in practice, would reduce the accuracy of the prediction. While the full historical demographic explanations for this distinction remain to be resolved, it is clear that the genomes of individuals with full Ashkenazi Jewish ancestry carry an unambiguous signature of their Jewish heritage, and this seems more likely to be due to their specific Middle Eastern ancestry than to inbreeding.

## Materials and methods

### Self-described race/ethnicity

The subjects were initially asked to check one of the following racial/ethnic labels: African, African-American, Caucasian, Hispanic, Native American, Middle Eastern/Central Asian, East Asian, South Asian, and Pacific Islander. They were also asked to write down the country of origin of all four grandparents. Following these categorizations the subjects were also asked if any of their grandparents were Jewish, and if so, to indicate which type (with Ashkenazi and Sephardic provided as examples). Only Caucasian subjects were selected for analysis, and country of origin data are provided for those subjects that had this information (Additional data file 1). The study was approved by the Duke University Medical Center IRB, and all subjects gave informed consent.

### Genotyping

All subjects were whole-genome genotyped using either the Illumina Infinium HumanHap550 version 1, the Illumina Infinium HumanHap550 version 3 or the Infinium HumanHap 610-quad chips. For quality control purposes, we employed a 'one percent rule' that discarded from analysis any SNP that had more than 1% of samples that could not be reliably scored, and any sample that had fewer than 99% SNPs reliably called. We carried out a test of cryptic relatedness to remove any relatives or duplicates within the sample, and we ensured that all genetically predicted sexes matched those in the genotype file, to check for genotype-ID mismatches. Genotype files are available as plink map and ped files (Additional data file 4).

### Eigenstrat analysis

To find Eigenstrat axes, we started with autosomal SNPs with minor allele frequency >0.01 from the set of SNPs that overlapped between the Illumina Infinium HumanHap550 version 1, Illumina Infinium HumanHap550 version 3, and the Infinium HD 610-quad chips. In order to prevent over-representation of regions with more redundant SNPs, we used the -- indep-pairwise command in the plink software package [11] to reduce linkage disequilibrium between the remaining variants by eliminating any SNP that had a pairwise r^2 ^> 0.3 with any other SNP in a 1,500 bp window (step size 150 bp). We also removed SNPs from certain known high LD regions (chr8:8000000...12000000, chr6:25000000...33500000, chr11:45000000...57000000, chr5:44000000...51500000). This reduced the original dataset to 121,834 SNPs (Additional data file 2). We then used smartpca and EIGENSOFT [8,12]. After the first run of smartpca there were 12 significant axes, but several of these seemed to be driven by a small number of outlier individuals. We then repeated the principal components analysis (PCA), this time allowing the automatic removal of all individuals that exceeded six standard deviations along the first ten principal component axes. Over 4 iterations, 19 outliers were removed, leaving 611 subjects. The 19 outliers included two subjects with 4 Jewish grandparents (1 identified by the subject as Sephardic), 1 with 2 Jewish grandparents and 16 of non-Jewish ancestry. In most cases the outliers had one or more grandparents from the Middle East, North Africa, Hawaii or South America.

### Statistical analysis of association of ancestry with principal component axes

PCA of the whole-genome data (after LD reduction) indicated the existence of five major ancestry axes, all driven by SNPs distributed throughout the genome. As expected from the work of Price *et al*. [7], the first principal component emerging from the Eigenstrat analyses was strongly correlated with Jewish ancestry (one-way ANOVA comparing groups with 0, 1, 2, 3 and 4 Jewish grandparents, F_4,606 _= 1068.4, *p *= 8.5 × 10^-273^). We therefore used an individual's position on this axis to assess how well Jewish ancestry could be predicted from the genetic data. Specifically, we used one-way ANOVA to see if those subjects declaring 1 (n = 8), 2 (n = 37), 3 (n = 4) or 4 (n = 55) Jewish grandparents were significantly different on PC1 from those declaring no Jewish grandparents (n = 507), and we identified clear clusters of individuals with different ancestries on this first principal component.

### Analysis on combined Duke and CEPH-HDGP samples

Firstly we attempted to analyze population structure on the mixed Duke/CEPH populations by including all SNPs, having again reduced redundancy as described above. However, because the Duke and CEPH populations were genotyped and quality controlled separately, a PCA that included all SNPs revealed multiple significant axes, reflecting systematic differences in laboratory procedures between the two genotyping sites. In order to focus on axes that reflected population structure and not genotyping procedures, we reduced the dataset to a core set of SNPs that did not show any systematic differences between the sites. To do this, we first reduced the dataset by sorting the reduced-LD dataset by chromosome and location and selecting a subset of SNPs (for example, one in every five along the chromosome) for analysis. We then removed any SNP that did not have genotype data for all individuals. We performed the PCA using EIGENSOFT as above, and examined the q-q plots for the distribution of SNP loadings, checking for axes with heavy tails. If such axes were detected, we removed the 'outlier' SNPs with particularly strong loadings on those axes (absolute value >1), and reapplied EIGENSOFT as before. Once all axes appeared to have a normal distribution, we plotted the axes and colored the subjects according to country of origin, and checked to see that the Duke population was mixed in with the European samples as expected (for example, subjects with four Italian or Russian grandparents clustered closer to their respective CEPH populations than to each other). We found that the largest set of SNPs that met these criteria was a set of 13,573 SNPs (Additional data file 3). It is possible that we could increase this dataset and gain better resolution for the European populations that remain undistinguished, but this was sufficient for the needs of this work.

## Abbreviations

PC1: first principal component axis; PCA: principal components analysis; SNP: single nucleotide polymorphism.

## Authors' contributions

ACN, ETC and DK recruited the subjects, genotyped them and performed genotyping quality control; ACN performed the analyses and ACN and DBG wrote the paper.

## Additional data files

The following additional data are available with the online version of this paper. Additional data file 1 is a CSV file displaying the country of origin (or ancestry if US was country of origin and ancestry was provided) of the 611 European-American samples used in the study. Additional data file 2 is a list of all SNPs included in the original analysis (n = 121,834). Additional data file 3 is a list of all SNPs included in the combined Duke and CEPH analysis (n = 13,573). Additional data file 4 is a zip file providing genotype data in plink format for the 121,834 SNPs included in the original analysis.

## Supplementary Material

Additional data file 1 'No data' is inserted if the subject identified the grandparents as Caucasian, Eurasian or American. Click here for file

Additional data file 2 SNPs included in the original analysis (n = 121,834). Click here for file

Additional data file 3 SNPs included in the combined Duke and CEPH analysis (n = 13,573). Click here for file

Additional data file 4 This includes a map file and a ped file. The map file has one line per SNP and the following columns: chromosome, rs#, genetic distance (blank) and position. The ped file has one line per subject and the following columns: Family ID (anonymous identifier), Individual ID (always 1, as subjects are unrelated), Paternal ID, Maternal ID (both zero), sex (1 = male, 2 = female) and phenotype, which in this case is the number of declared Jewish grandparents. Click here for file
